# Systematic Review: Dementia Post-Diagnostic Support in UK Rural Communities: Experiences of People Living With Dementia, Informal Caregivers, and Healthcare Professionals

**DOI:** 10.1192/bjo.2024.117

**Published:** 2024-08-01

**Authors:** Danielle Bilkey, Ellena Businge

**Affiliations:** 1 Cornwall Partnership NHS Trust, Truro, United Kingdom; 2 The University of Plymouth, Plymouth, United Kingdom; 3 The University of Exeter, Exeter, United Kingdom

## Abstract

**Aims:**

The aim of this systematic review is to identify and describe the experiences, barriers and provision of post-diagnostic support in UK rural areas; from the perspective of people living with dementia, healthcare professionals and informal family caregivers.

**Background:**

People living with dementia in rural areas experience numerous barriers to accessing post-diagnostic support.

**Methods:**

Systematic Review.

Systematic searches will be conducted in the following databases; SCOPUS, PubMed, PsychINFO and CINAHL Plus. Systematic review tool Rayyan.ai will be used to screen titles and abstracts, prior to full-text review. Following data extraction, The Critical Appraisal Skills Programme (CASP) tool will be used to appraise the quality of studies and assess risk of bias. The data will be deductively analysed through the lens of the Candidacy Framework's 6 dimensions, with a secondary inductive analysis capturing any themes that fall outside of the framework.

**Results:**

242 papers have been screened by first and second reviewer. 15 papers included. Papers still being analysed for full review. Will be complete by March 2024.

**Conclusion:**

This systematic review will help improve understanding of the rural barriers and experiences of post-diagnostic support, and allow researchers and stakeholders to develop and optimise specially tailored dementia interventions in line with the needs of people residing in UK rural communities.

First reviewer: Danielle Bilkey, Second Reviewer: Dr Ellena Businge, Supervisor: Dr Nicolas Farina.

